# Genetic characterization and genome-wide association mapping for dwarf bunt resistance in bread wheat accessions from the USDA National Small Grains Collection

**DOI:** 10.1007/s00122-020-03532-0

**Published:** 2020-01-14

**Authors:** Tyler Gordon, Rui Wang, David Hole, Harold Bockelman, J. Michael Bonman, Jianli Chen

**Affiliations:** 1 USDA-ARS-Small Grains and Potato Germplasm Research Unit, 1691 S. 2700 W., Aberdeen, ID 83210 USA; 2 University of Idaho-Aberdeen Research and Extension Center, 1693 S. 2700 W., Aberdeen, ID 83210 USA; 3 Department of Plants, Soils and Climate, Utah State University, 2325 Old Main Hill, Logan, UT 84322 USA

## Abstract

**Key message:**

Dwarf bunt-resistant bread wheat accessions and SNP markers associated with DB resistance identified in this study are valuable resources for characterization and deployment of DB resistance in bread wheat.

**Abstract:**

Dwarf bunt (DB), caused by *Tilletia controversa* J.G. Kühn, can significantly reduce grain yield and quality on autumn-sown wheat in regions with prolonged snow cover. DB can be managed with the use of resistant cultivars. The objectives of the present study were to characterize DB resistance in a large set of bread wheat accessions from the National Small Grains Collection and use a genome-wide association study approach to identify genetic loci associated with DB resistance. A total of 292 accessions were selected using historical DB resistance data recorded across many trials and years in the Germplasm Resources Information Network (GRIN) and re-tested for DB resistance in replicated field nurseries in Logan, UT, in 2017, 2018, and 2019. Ninety-eight accessions were resistant with DB normalized incidence ≤ 10%, and twenty-eight of these were highly resistant with DB normalized incidence ≤ 1% in both GRIN and the field nurseries. Based on the presence of marker haplotypes of the four published dwarf bunt QTL on 6DS, 6DL, 7AL, and 7DS, highly resistant accessions identified in this study may provide novel resistance and should be further evaluated. This study validated one previously identified QTL on 6DS and identified an additional locus on 6DS. These loci explained 9–15% of the observed phenotypic variation. The resistant accessions and molecular markers identified in the present study may provide valuable resources for characterization and deployment of DB resistance in bread wheat.

**Electronic supplementary material:**

The online version of this article (10.1007/s00122-020-03532-0) contains supplementary material, which is available to authorized users.

## Introduction

Bread wheat (*T. aestivum* L.) is an important food staple and 772 million t were harvested globally in 2017 (FAOSTAT 2019). Dwarf bunt (DB), caused by the basidiomycete *Tilletia controversa* J.G. Kühn [as *‘contraversa*’] in L. Rabenhorst (Kühn 1874), and common bunt (CB), caused by two closely related fungi *Tilletia caries* (DC.) Tul. & C. Tul. [syn. *T. tritici* (Bjerk.) G. Wint.] and *Tilletia laevis* J. G. Kühn [syn. *T. foetida* (Wallr.) Liro], are destructive diseases of bread wheat and durum wheat (*T. turgidum* subsp. *durum* Desf.) (Goates 1996). While these three pathogens vary slightly in their spore morphology and etiology, they are closely related with similar modes of infection and means of control. DB and CB differ slightly; in that *T. controversa* infects autumn-sown wheat and requires several months of snow cover for teliospore germination on the soil surface, whereas *T. caries* and *T. laevis* primarily infect spring-planted wheat from spores in the soil.

Initiation of DB and CB begins when dikaryotic infection hyphae penetrate emerging seedlings thereby infecting the developing apical meristem (Kollmorgen and Ballinger 1987). The resulting systemic infection is often cryptic until flowering, when the fungal hyphae invade and replace developing ovaries with darkly pigmented teliospores that comprise a fungal sorus or bunt ball (Goates 1996; Castlebury et al. 2005). Yield losses due to DB and CB can exceed 80%, and trimethylamine emitted by the teliospores causes a fetid, rotting fish odor which reduces flour quality (Goates 1996; Castlebury et al. 2005).

Difenoconazole, a seed treatment fungicide, effectively controls both diseases without causing yield reductions or phytotoxicity (Keener et al. 1995; Goates 1996) though genetic resistance offers a cost-effective compliment to seed treatments particularly in organic production systems. Most of the wheat landraces in the USDA National Small Grains Collection (NSGC) were screened for bunt resistance over the past 30 years, and resistance was found primarily in germplasm originating from regions in Iran, Macedonia, Montenegro, Serbia, and Turkey (Bonman et al. 2006). However, resistance was relatively rare. Among 10,759 landrace accessions tested for CB resistance, only 597 (5.5%) were resistant, and of 8,167 landrace accessions tested for DB resistance, only 104 (1.3%) were resistant (Bonman et al. 2006).

DB and CB resistance is putatively controlled by gene-for-gene interactions, and it is assumed that the same genes confer resistance to both diseases (Hoffman and Metzger 1976; Goates 2012). An expanded set of bunt differential wheat accessions representing 16 *Bt* genes was developed to elucidate host–pathogen interactions (Goates 2012). Using these *Bt* differentials, Goates (2012) found 19 pathogenic races of *T. controversa*, 36 races of *T. caries*, and 15 races of *T. laevis,* and determined that *Bt8* (PI 554120), *Bt11* (PI 554119), and *Bt12* (PI 119333) were broadly effective against most races of DB and CB.

Genomic tools in wheat including dense molecular marker arrays with annotations (Wang et al. 2014), genotyping by sequencing, and reference genome sequences (IWGSC 2018) have enabled the identification of genetic loci underpinning DB and CB resistance (Supplementary File 1). Linkage mapping (Chen et al. 2016; Singh et al. 2016; Steffan et al. 2017) and association mapping techniques (Bhatta et al. 2018; Mourad et al. 2018) have located bunt resistance loci on 19 wheat chromosomes. Identifying markers tightly linked to resistance will enable the discovery of additional resistance genes and introgression of multiple resistance genes into adapted cultivars.

The NSGC is a worldwide collection of the small grains and contains 42,544 bread wheat accessions. Of these, 19,378 accessions have been systematically characterized for DB resistance since the early 1980s, and only 129 (0.7%) are classified as resistant based on a DB incidence threshold of ≤ 10% proposed by Goates (2012). The purpose of this study was to: 1) verify the DB resistance in the NSGC bread wheat accessions with replicated field trials, and 2) identify genetic loci associated with DB resistance using a genome-wide association study (GWAS) approach.

## Materials and methods

### Plant materials

DB resistant and susceptible accessions were selected for this panel based on data from the US National Germplasm System online database: Germplasm Resources Information Network (GRIN), accessed at https://npgsweb.ars-grin.gov/gringlobal/search.aspx. Using a resistance threshold of ≤ 10% disease incidence relative to the susceptible check (Goates 2012), only 129 GRIN accessions were classified as DB resistant. An additional seven accessions with DB incidence below 13% were also included in the panel for a total of 136 bread wheat accessions classified as resistant for the GWAS. In an attempt to mitigate the effects of population structure on the GWAS, one susceptible accession from the same geographic region as each resistant accession was selected. For example, PI 470452 was classified as resistant and originated in Agri Province, Turkey; therefore, a susceptible accession from Agri Province, Turkey, PI 470470, was also selected. Additionally, the bunt differentials (Goates 2012), including *Bt0* through *Bt15*, *Btp*, and PI 173438 (unknown *Bt*), and two known susceptible winter cultivars ‘Wanser’ (CItr 13844) and ‘Cheyenne’ (CItr 8885), were also included in the GWAS panel. Supplementary File 2 lists the accession number, name, taxon, geographic origin, improvement status, pedigree, and DB incidence for each of the 292 accessions.

### Field trials

Since the 1980s, GRIN DB normalized incidence (NI) relative to the susceptible check cultivar ‘Cheyenne’ in each trial was collected from NSGC accessions grown at the Green Canyon USDA-ARS disease screening nursery in Logan, UT (approximately 3 km east of Logan: 41°46′21.05″N, 111°46′52.68″W, elevation 1450 m). DB field trials conducted in 2017, 2018, and 2019 were evaluated near the Green Canyon site at the Utah State University (USU) Research Farm in Logan, UT (41°45′46.46″N, 111°48′54.98″W, elevation 1400 m). USU field trials were sown with a head row planter on October 10, 2016, September 27, 2017, and September 18, 2018 with one accession per 1-m row and two replications in 2017 and 2018 and one replication in 2019. Each row was inoculated after seedling emergence on November 4, 2016, October 24, 2017, and November 6, 2018, with approximately 100 ml of a concentrated DB teliospore suspension (2 × 10^6^ spores ml^−1^ water). A composite of teliospores from infected spikes previously collected in the USU DB nursery were used for the inoculations. Disease incidence was assessed on fully mature adult plants, Zadoks stage 94 (Zadoks et al. 1974), on August 3, 2017, July 26, 2018, and August 6, 2019 by counting the number of spikes where at least one floret was infected, and dividing by the total number of spikes in the row. DB incidence per replicate was normalized to the average of the six plots of the susceptible cultivar, Wanser. Accession DB incidence and NI are reported in Supplementary File 2, and the mean DB NI for each field trial can be accessed through GRIN.

### Molecular marker assessment

A modified DNA CTAB protocol was used to extract genomic DNA from seedlings at the 2–3 leaf stage (Babiker et al. 2015). A 2-cm segment of leaf tissue was placed into 96 well Corning^®^ Costar^®^ tubes (Corning, NY, USA) and macerated in CTAB extraction buffer with a bead grinder. The aqueous layer was separated in chloroform, extracted, and the precipitate was washed with isopropanol and then ethanol. Resulting DNA pellets were suspended in Tris (10 mM) and sent to the USDA-ARS Small Grains Genotyping Laboratory in Fargo, ND, where samples were genotyped using the 90 K iSelect SNP assay as described by the manufacturer (Illumina, San Diego, CA). Allele clustering was completed using Genome Studio v.2.0.2 (Illumina), and the resulting set of 41,511 polymorphic SNPs was exported to JMP Genomics v.9.0 (SAS Institute Inc., Cary, NC, USA) for filtering. Markers were excluded if minor allele frequency (MAF) was < 4%, or missing data were > 10%. Heterozygous calls were also removed. Accessions were classified as duplicates and removed if they were ≥ 99.7% identical across all polymorphic SNPs. A final group of 246 bread wheat accessions were selected, and 19,281 SNP markers were aligned with the physical wheat annotation (IWGSC 2018) and used for subsequent marker-trait associations.

### Statistical analyses

Unless stated otherwise, all statistical analyses were conducted using JMP^®^ Genomics v. 9.0. By design, the DB NI had a bimodal distribution, and a Shapiro–Wilk normality test (Shapiro and Wilk 1965) of trial residuals indicated a significant (*P* < 0.0001) shift from normality. Similarly, square root and log_10_ transformations of the trials indicated significant (*P* < 0.0001) deviations from normality, and the untransformed DB NI data were used in all further analyses. A mixed model with genotype set as a fixed effect and trial as a random effect was used to calculate best linear unbiased estimates (BLUEs) for DB NI across trials and replications (Henderson 1975). Broad-sense heritability (H^2^) was calculated using the formula: $H^{2} = \, \sigma_{G}^{2} /\left[ {\sigma_{G}^{2} + \, \sigma_{ExG}^{2} /r + \, \sigma_{2}^{2} /r} \right]$ where $\sigma_{G}^{2}$ is the genotypic variance, $\sigma_{ExG}^{2}$ is the interaction variance between trial and genotype, $\sigma_{e}^{2}$ is the residual variance, and r is the number of data sets (Hanson et al. 1956). Correlation coefficient estimates between trials were calculated using a Spearman’s Rho nonparametric rank-sum correlation procedure.

Genome-wide linkage disequilibrium (LD) was calculated as *r*^2^ values between each marker within chromosome groups (Supplementary File 3). An IBS familial relationship matrix (k matrix) and heat map were generated using the Ward hierarchical clustering method (Ward Jr and Hook Ward and Hook 1963) to explore potential subpopulations within the panel. STRUCTURE v.2.3.4 (Pritchard et al. 2000) and STRUCTURE HARVESTER (Earl and vonHoldt 2012) software packages were used to optimize the number of subpopulations (k). In STRUCTURE, the burn-in iterations and Markov chain Monte Carlo replications were set to 10,000, the admixture correlated model was selected, and five replicate iterations were performed. Proposed subpopulations with k between 1 and 10 were evaluated in STRUCTURE HARVESTER using the Evanno method (Evanno et al. 2005), and the number of subpopulations that corresponded with the highest Δk value was selected as the optimal model.

A principal component analysis with ten principal components (PCs) was generated (Q matrix) to explore population stratification, and the resulting scree plot was used to estimate the optimal number of PCs that would explain the most variation in the models (Price et al. 2006). Bayesian information content (BIC) assessments (Burnham and Anderson 2004) were used to formally test the various association analysis models. Tested models included a general linear model (GLM) without corrections for K or Q (the naïve model), a GLM that corrected for population stratification with 2, 3 or 5 PCs, and mixed linear models (MLMs) that controlled for both familial relationships, as a random effect, and population stratification with 2, 3 or 5 PCs as a fixed effect (Yu et al. 2006). All models correcting for familial relationships performed better than the Naïve model. A MLM with a kinship covariate matrix and two PCs had the lowest BIC value and was therefore chosen for further marker-trait association analysis.

Marker-trait associations between DB NI and SNP markers were conducted on trial means, and BLUEs. Resulting *P* values were adjusted using an FDR multiple testing procedure (Benjamini and Hochberg 1995), and a significance threshold of *P* ≤ 0.05 on FDR-adjusted *P* values was used to identify SNP-trait associations for further analysis. SNPs significantly associated with DB NI in any trial or BLUE were aligned with the Chinese Spring reference genome sequence v1.0 (IWGSC 2018) using IWGSC BLAST (Alaux et al. 2018) with the highest coverage and identity location available. To assess potentially linked SNPs, the most significant marker in each putative marker-trait association group was included one at a time in the MLM as covariates. Markers in high LD with the covariate marker were no longer significantly associated with DB resistance and were grouped with the covariate SNP group.

## Results

### Field trials

Two susceptible check cultivars, Cheyenne and Wanser, showed a high incidence of DB in both USU field trials. The mean DB incidence for Wanser was 63.9% in 2017, 82.8% in 2018, and 67.3% in 2019, while Cheyenne had a mean DB incidence of 79.2% in 2017, 84.6% in 2018, and 88.6% in 2019. Across the three trials, all differentials showed consistent responses except for the *Bt9* differential, which was classified as resistant in 2017 but susceptible in 2018 and 2019, and the *Bt5* differential which was classified as susceptible in 2017 and 2019 but resistant in 2018 (Table 1).

**Table 1:** Bunt differential lines and known susceptible and resistant sources showing subpopulations, and dwarf bunt normalized incidence from the germplasm resources information network (GRIN), 2017, 2018, and 2019 Logan, UT field trials, and best linear unbiased estimates (BLUEs)

Accession	Name	Bt gene	Subpopulation	GRIN	2017	2018	2019	BLUE
CItr 8885	Cheyenne	Susceptible	4	100	119.1	102.2	131.6	116.2
CItr 13844	Wanser	Susceptible	4	100	100	100	100	104.4
PI 209794	Heins VII	*Bt0*	1	97	135.3	85.2	130.2	111.2
PI 554101	Selection 2092	*Bt1*	1	.	103.3	92.1	101.7	104.0
PI 554097	Selection 1102	*Bt2*	1	.	121.6	98.1	131.4	119.2
CItr 6703	Ridit	*Bt3*	3	76	10.5	42.6	67.2	51.2
PI 11610	CI 1558	*Bt4*	3	100	150.2	98.9	130.4	120.4
CItr 11458	Hohenheimer	*Bt5*	1	.	69.4	3.4	26.7	32.1
CItr 10061	Rio	*Bt6*	3	.	132.0	54.5	144.1	67.4
PI 554100	Selection 50077	*Bt7*	1	100	150.0	93.2	103.7	112.7
PI 554120	M72-1250	*Bt8*	2	.	0	3.0	1.9	7.5
PI 554099	R63-6968	*Bt9*	2	.	0	44.0	111.1	55.3
PI 554118	R63-6982	*Bt10*	2	.	17.9	19.0	69.3	37.0
PI 554119	M82-2123	*Bt11*	2	1	0	1.2	2.2	4.5
PI 119333	1696	*Bt12*	6	0	0	0	0	3.4
PI 181463	Thule III	*Bt13*	5	15	2.7	9.6	0.9	11.0
CItr 13711	Doubbi	*Bt14*	.	.	.	0.0	2.8	3.7
CItr 12064	Carleton	*Bt15*	.	.	.	9.6	15.5	14.8
PI 173437	7838	*Btp*	6	0	.	0.7	0	0.1
PI 173438	7845	Unknown	6	0	.	0	0.9	0.1
PI 178383	6256	*Bt8, 9, 10*	6	0	0	2.1	0	4.5
PI 476212	SM Selection 4	Unknown	6	1	0	0	0	4.0

**Fig. 1:**
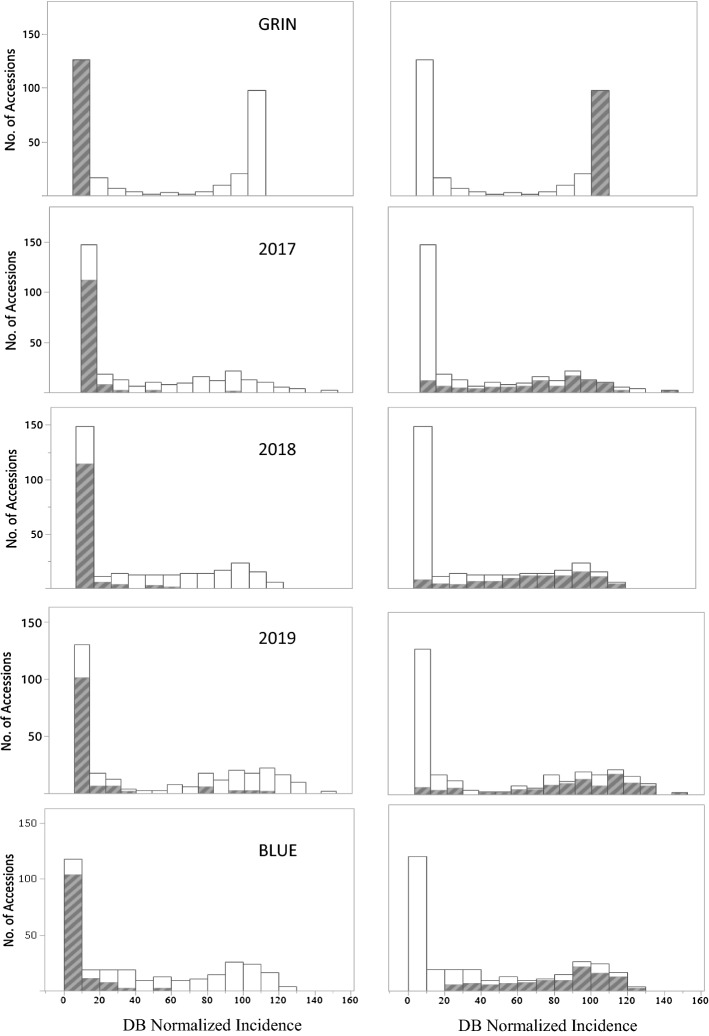
Dwarf bunt normalized incidence distributions across 292 wheat accessions from four data sets including the germplasm resources information network (GRIN), mean 2017, 2018, and 2019, Logan, UT field trials, and best linear unbiased estimates (BLUEs) from across trials; left pane: shaded accessions with DB normalized incidence ≤ 10% in GRIN, right pane: shaded accessions with DB normalized incidence ≥ 90% in GRIN

A mixed model ANOVA (Supplementary File 3) found no significant trial effect, but there was a significant genotype and genotype-by-trial effect (*P* < 0.0001). Broad-sense heritability (H^2^) for DB NI was estimated at 0.93. Best linear unbiased estimates derived from the mixed model of DB NI across trials are listed in Supplementary File 2. By design, the field trials were composed of approximately 50% resistant and 50% susceptible accessions as classified based on GRIN data (Fig. 1). USU field trials produced a similar response, with 50.3% showing resistance in 2017, 50.7% showing resistance in 2018, and 45.2% with resistance in 2019 (Table 2). Accessions classified as susceptible based on GRIN showed a wide array of disease incidence in the field trials (Fig. 1). Most of the accessions classified as resistant based on GRIN data, yet susceptible in 2017, 2018, or 2019, were breeding lines from the USA. Another group of accessions, about half being landraces from Turkey, showed the opposite reaction; they were susceptible based on GRIN data, but resistant in the USU field trials (Supplementary File 2).

**Table 2:** Number and percent of resistant and susceptible bread wheat accessions in four data sets and BLUEs and number of accessions that were consistent for resistance or susceptibility across all data sets

Data set^a^	Resistant^b^	Susceptible^c^	Percent resistant (%)
GRIN	128	162	44.1
2017	146	144	50.3
2018	147	143	50.7
2019	131	159	45.2
BLUE	116	174	40.0
Consistent across all trials	98	116	45.8

^a^Data sets including the germplasm resources information network (GRIN), mean 2017, 2018, and 2019 Logan, UT field trials, and best linear unbiased estimate (BLUE) from across trails

^b^Resistance classified as DB normalized incidence≤ 10%

^c^Susceptibility classified as DB normalized incidence> 10%

A Spearman’s rank-sum nonparametric correlation was used to measure the degree of similarity between and among the GRIN, 2017, 2018, and 2019 USU field trial means and BLUEs. Correlation coefficients (*r*^2^) between data sets ranged from 0.70 and 0.93, and all estimates were significant at *P *< 0.0001. GRIN was correlated with the 2017, 2018, and 2019 trials, and BLUEs with correlation coefficients of 0.76, 0.77, 0.70, and 0.85, respectively. The correlation coefficient between the 2017, 2018, and 2019 USU trials was 0.88, 0.76, and 0.78, respectively. In the 2017 USU field trials, the *r*^2^ between replications was 0.86, and in 2018, the *r*^2^ was 0.87 between the two replications.

There were 98 accessions that were resistant with a DB NI ≤ 10% across GRIN and 2017, 2018, and 2019 USU trial means (Supplementary File 2). Of these, 28 were highly resistant with a DB NI ≤ 1% across all trials (Table 4). These highly resistant accessions included eight Turkish landraces, and 14 US lines with Turkish landraces in their pedigree. The remaining six highly resistant accessions were landraces from Serbia (1), Montenegro (1), Iran (1), and three breeding lines from the USA (Table 3).

**Table 3:** Geographic origin and number of bread wheat accessions highly resistant, resistant, and susceptible to dwarf bunt (DB) across all data sets with the number of landraces within each group shown in parenthesis

Accession Origin	DB resistance category		
Country	HR^a^	R^b^	S^c^
Azerbaijan	0	0	3 (2)
Germany	0	0	1
Iran	1 (1)	8 (8)	10 (10)
Montenegro	1 (1)	1 (1)	2 (2)
Russia	0	1	1 (1)
Serbia	1 (1)	6 (6)	9 (9)
Spain	0	0	1 (1)
Turkey	8 (8)	26 (25)	17 (13)
USA	17	56	72
Total	28	98	116

^a^Highly resistant accessions with a DB NI ≤ 1%

^b^Resistant accessions with a DB NI ≤ 10%

^c^Susceptible accessions with a DB NI > 10%

### Population structure

There were 44 accessions that were ≥ 99.7% identical across the 19,281 SNPs. These duplicate and near-duplicate accessions originated from similar geographic regions and had similar DB NI across data sets (Supplementary File 2), and were removed for further analyses. Genetic similarity among the 246 non-duplicated accessions ranged from 53% to 99% with a mean similarity of 67%.

**Fig. 2:**
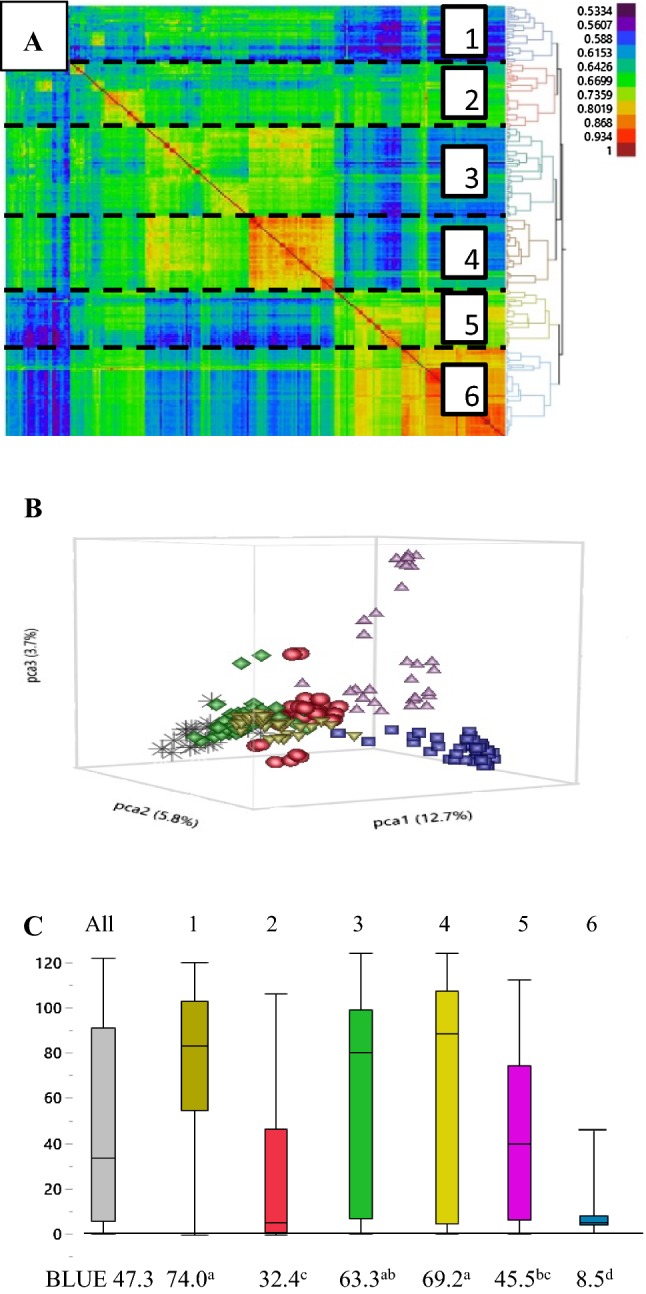
**a** Genetic similarity heat map derived from an identity-by-state relationship matrix of 246 by 246 bread wheat accessions, regions of high (red) and low (purple) similarity between accessions; and a dendrogram showing six subpopulations (1–6) each separated by a dashed line. **b** Accessions plotted with three principal components showing subpopulations: 1 (brown stars), 2 (red circles), 3 (green diamonds), 4 (brown triangles), 5 (purple triangles), 6 (blue squares). **c** Best linear unbiased estimate (BLUE) dwarf bunt normalized incidence quantile box plots, left to right: all 246 accessions (gray) and subpopulations: 1 (blue), 2 (red), 3 (green), 4 (yellow), 5 (purple), 6 (blue); mean BLUE values are listed for each subpopulation below their respective box plots, means followed by a common letter are not significantly different by Tukey’s HSD at *P* ≤ 0.05 (color figure online)

Based on the STRUCTURE HARVESTER Δk values, there were six distinct subpopulations (k = 6) in the panel, and these groupings were supported by visual assessment of the Ward hierarchical clustering heat map and principal component analysis (Fig. 2a, b). Subpopulations based on the marker data corresponded well with geographic origin (Table 4). Subpopulation 1 and 4 consisted primarily of breeding lines and cultivars from the USA. Subpopulation 2 consisted of accessions from Turkey and breeding lines from the USA. Serbian landraces predominated in subpopulation 3, while landraces from Iran were primarily located in subpopulation 5. Landraces from Hakkari province, Turkey, and breeding lines from the USA that had Turkish landraces in their pedigree, were grouped into subpopulation 6 (Table 4). The bunt differentials were distributed across all the subpopulations (Table 1).

**Table 4:** Geographic origin and number of bread wheat accessions in each subpopulation

Accession origin	Subpopulation					
Country	1	2	3	4	5	6
Azerbaijan	0	0	1	0	2	0
Germany	1	0	0	0	0	0
Iran	0	5	0	0	19	3
Montenegro	1	0	2	1	0	0
Russia	0	0	1	0	1	0
Serbia	2	1	15	0	0	0
Spain	1	0	0	0	0	0
Sweden	0	0	0	0	1	0
Turkey	4	25	1	0	11	42
USA	24	16	31	43	0	36
Total	33	47	51	44	34	81

The BLUE DB NI estimate for the entire panel was 47.3% (Fig. 2c), and BLUE values for each subpopulation differed significantly at *P* < 0.0001. Subpopulation 6 had the lowest mean BLUE DB NI of 8.5%, and subpopulation 1 had the highest mean BLUE DB NI of 74.0%. Of the 98 accessions that were resistant across trials (Table 3), 7% were in subpopulation 6, 13% were in subpopulation 2, with the remainder in subpopulations 1, 3, 4, and 5. Of the 28 highly resistant accessions, 75% were in subpopulation 6, 7% were in subpopulation 2 and 3, and 4% were in each of the subpopulations 1, 4, and 5.

### Linkage disequilibrium

Genome-wide marker-pair *r*^2^ correlations between 19,281 SNPs were plotted as a function of intrachromosomal inter-marker genetic distance (Supplementary File 4). A median *r*^2^ of 1 was found between SNP markers that were completely linked with an inter-marker physical distance of 0 Mbp. LD median *r*^2^ decreased to 0.1 at an inter-marker distance of 0.1 to 1 Mbp indicating an LD decay rate of 90% over the 1 Mbp interval.

A smoothing spline curve with lambda equal to 10,000 was fit to the LD scatter plot to determine a genome-wide QTL confidence interval (Supplementary File 4). Others (Maccaferri et al. 2015; Liu et al. 2017) have used an LD of *r*^2^ = 0.3 as a threshold for genome-wide QTL confidence intervals in wheat. In the present study, the largest spline curve *r*^2^ value was 0.45. When the smoothing spline curve was set to *r*^2^ = 0.3, the physical distance was 0.67 Mbp, and when the curve was set to *r*^2^ = 0.1, the distance was 6.80 Mbp.

### Marker-trait associations

**Fig. 3:**
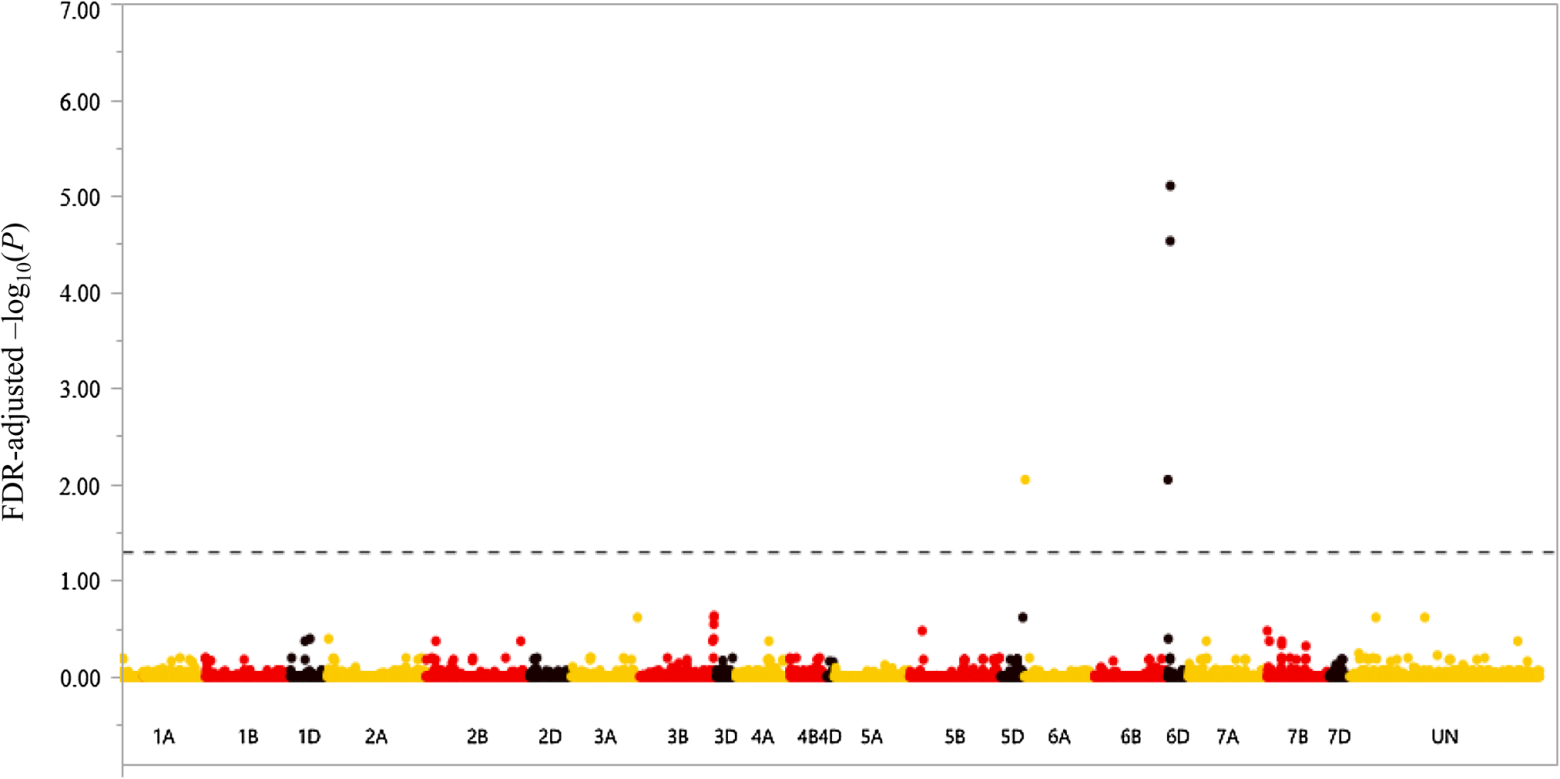
Manhattan plot showing associations between 19,281 SNP markers and dwarf bunt normalized incidence best linear unbiased estimates (BLUEs) across 246 bread wheat accessions; the horizontal dashed line indicates an FDR-adjusted significance threshold of *P* = 0.05; A-, B- and D-genome SNP markers are represented by yellow, red, and black dots, respectively (color figure online)

After controlling for kinship and population stratification, GWAS revealed four SNPs significantly (FDR-adjusted *P *< 0.05) associated with DB incidence in at least one trial or BLUE (Table 5, Fig. 3, and Supplementary Files 5, 6, 7 and 8). FDR-adjusted negative log_10_*P* values for BLUE DB NI from these six marker-trait association groups ranged from 1.7 to 5.1, phenotypic variance (*r*^2^) ranged from 0.09 to 0.15, and average DB NI BLUE values for accessions carrying resistance alleles ranged from 16.1 to 40.8 (Table 5). One marker-trait association group represented by two SNPs on chromosome 6DS was significant in three of the data sets (Table 5). Marker-trait association groups aligned with the 246 bread wheat accessions used for the GWAS (Supplementary File 9) show a corresponding decrease in DB NI as the number of resistant allele haplotypes increases (Supplementary File 10).

**Table 5:** Marker-trait association groups significantly (FDR-adjusted *P* ≤ 0.05) associated with dwarf bunt (DB) resistance in the panel of 246 bread wheat accessions in at least two data sets from the germplasm resources information network (GRIN), mean 2017, 2018, and 2019, Logan, UT field trials, or best linear unbiased estimates (BLUEs) across trials

DB Marker-trait group	Chr.^a^	QTL Range (Mbp)^b^	SNP Index^c^	Additional markers^d^	SNP^e^	FDR-adjusted negative log_10_(*P*)	*r*^2^	RAF^f^	R SNP^g^	S SNP^g^	Significant data sets
*DB*-*6D1*	6D	1.77	IWB21614	1	[T/**C**]	1.8	0.09	0.85	40.8	87.0	BLUE
*DB*-*6D2*	6D	6.97 to 7.29	IWB59793	1	[A/**G**]	5.0	0.15	0.14	16.1	52.2	GRIN, 2018, BLUE

^a^Physical chromosome locations of each marker-trait association group with 99 or 100% identity based on the physical annotation of wheat (IWGSC 2018)

^b^Physical regions in Megabase pairs (Mbp) based on the physical annotation of wheat (IWGSC 2018)

^c^Single nucleotide polymorphism (SNP) 90 K index according to Wang et al. 2014 in each marker-trait association group with the lowest *P* value; additional associated SNPs are reported in Supplementary file 3

^d^Additional SNPs in the marker-trait association group in high LD (≥ 0.8) with the SNP index

^e^SNP with resistance allele in bold and underlined

^f^Resistance allele frequency (RAF) for each indicator SNP

^g^Average DB normalized incidence values associated with the resistant (R) and susceptible (S) SNP allele; a low value indicates a high level of resistance

## Discussion

Uniform DB infection requires specific environmental conditions that include several weeks of stable cool soil temperatures, a moist environment at the soil surface, and low light levels. These conditions are most reliably provided by continuous snow cover and are critical for teliospore germination (Chen et al. 2016). The two susceptible check cultivars, Wanser and Cheyenne, showed high DB incidence in all field trials indicating that the environmental conditions favored infection by the DB pathogen.

There were 28 highly resistant accessions with a DB NI ≤ 1% across all data sets. Twenty-one of these highly resistant accessions either originated in Turkey or have Turkish landraces in their pedigrees. Similarly, the four bunt differentials that were highly resistant across trials, PI 554119 (*Bt11*), PI 119333 (*Bt12*), PI 173437 (*Btp*), and PI 173438 (unknown Bt), all either originated in Turkey or had a Turkish landrace in their pedigree. PI 119333 and PI 173437 had similar haplotype profiles (Table 6), and they shared SNP marker haplotypes with some of the other highly resistant accessions (Supplementary File 9). For instance, PI 119333 (*Bt12*) shares a similar haplotype profile to six other highly resistant accessions, and PI 173438 (with unknown *Bt*) shares a similar profile with two other highly resistant accessions including PI 476212 (Table 6).

**Table 6:** Highly resistant landrace accessions with dwarf bunt (DB) normalized incidence ≤ 1% across all data sets, bunt differentials and several known resistant and susceptible accessions with corresponding best linear unbiased estimates (BLUE) values and the presence (+) or absence (−) of the resistant allele haplotypes from each marker-trait association group detected in this study

Accession	Bt gene	Origin	BLUE	*DB*-*6D1*	*DB*-*6D2*	*QDB.ui*-*6DL*^*a*^	*QDB.ui*-*7AL*^*b*^	*QDB.ui*-*7DS*^*c*^
PI 345106		Serbia	3.9	+	−	−	−	−
PI 345428		Montenegro	4	−	−	−	−	−
PI 476212		USA	4	+	−	−	−	+
PI 560601		Turkey	3.8	+	−	−	−	−
PI 560602		Turkey	3.8	+	−	−	−	−
PI 560842		Turkey	3.8	+	−	−	−	+
PI 560843		Turkey	3.8	+	−	−	−	−
PI 560848		Turkey	3.8	+	−	−	−	+
PI 627677		Iran	4.1	+	−	−	−	−
CItr 8885	Susceptible	USA	116.2	−	−	−	−	−
PI 209794	Susceptible	Germany	111.2	+	−	−	−	−
PI 554101	*Bt1*	USA	104	−	−	−	−	−
PI 554097	*Bt2*	USA	119.2	−	−	−	−	−
CItr 6703	*Bt3*	USA	51.2	+	−	−	−	−
PI 11610	*Bt4*	USA	120.4	+	−	−	−	−
CItr 11458	*Bt5*	USA	32.1	+	−	−	−	−
CItr 10061	*Bt6*	USA	67.4	+	+	−	−	−
PI 554100	*Bt7*	USA	112.7	−	−	−	−	−
PI 554120	*Bt8*	USA	7.5	+	−	−	−	−
PI 554099	*Bt9*	USA	55.3	−	−	+	−	−
PI 554118	*Bt10*	USA	37	+	+	−	−	−
PI 554119	*Bt11*	USA	4.5	+	−	−	−	−
PI 119333	*Bt12*	Turkey	3.4	+	−	−	−	+
PI 181463	*Bt13*	Sweden	11	+	−	−	−	−
PI 173437	*Btp*	Turkey	0.1	+	−	−	−	−
PI 173438	*Bt* (unknown)	Turkey	0.1	+	−	−	−	+
PI 178383	*Bt8,9,10*	Turkey	4.5	+	+	+	−	−

^a^The 6DL haplotype SNP markers are reported in Wang et al. (2019)

^b^7AL haplotype SNP markers are reported in Wang et al. (2019)

^c^7DS haplotype SSR markers are reported in Chen et al. (2016), and SNP markers were reported by Rui Wang (personal communication)

Based on the pedigree analysis (Supplementary File 2), many highly resistant breeding lines are derived from resistant Turkish landraces. PI 178383 and PI 476212 are in the pedigrees of several DB resistant cultivars, such as ‘Weston,’ ‘DW,’ ‘Golden Spike,’ and ‘UI Silver’ (Hole et al. 2002). However, some highly resistant landraces PI 345106 from Serbia, PI 345428 from Montenegro, and PI 627677 from Gilan province, Iran, have unique haplotypes and geographic origins (Table 6). Therefore, mapping the DB resistance within these unexploited resistance sources is an important step toward future molecular breeding for DB resistance.

In the present study, accessions were selected based on a DB NI resistance threshold of ≤ 10%. Other accessions with intermediate levels of resistance are of interest to geneticists and plant breeders as they may contain a complex of minor or partial resistance genes. Specifically, PI 362710 from Montenegro, PI 345480 from Serbia, and PI 636153 a breeding line from Idaho, USA, had intermediate levels of DB resistance across data sets. Additionally, in the GRIN database, there are 976 bread wheat accessions that have a DB incidence recorded between 11 and 30%. Environmental conditions can make bunt disease incidence variable from one year to the next. Thus, to confirm the partial resistance that may exist in these accessions, more research is warranted. Single-seed derived lines of each accession could be tested for multiple years in the field. Alternatively, molecular marker-assisted evaluation could be undertaken to identify accessions that do not carry known resistance QTL haplotypes. A quantitative PCR assay, like those developed for rust diseases (Admassu-Yimer et al. 2019), that reliably measures the degree of tissue colonization by the bunt pathogen could also provide a means for assessing partial resistance to the disease under greenhouse conditions.

Six subpopulations were selected in this panel of 246 bread wheat accessions based on Δk value optimization using STRUCTURE and STRUCTURE HARVESTER. These six subpopulations roughly corresponded to the geographic origins listed in GRIN (Table 5). We attempted to control for population relatedness by selecting both resistant and susceptible accessions from the same geographic area. Unfortunately, the subpopulations differed significantly in their levels of DB incidence (Fig. 2c) which could affect marker-trait associations. Specifically, those accessions in subpopulation 6 which corresponded with a Hakkari province, Turkey origin, had significantly lower DB NI values than the other five subpopulations. Investigators may need to limit the origin of accessions to one region or locality to better balance population structure when designing future bunt GWAS. For instance, it might be of interest to examine all landrace accessions from Turkey as one study, and all landrace accessions from Iran as a separate study.

Broad-sense heritability, 0.93, was high for DB NI in this panel. Others have also reported high broad-sense heritability estimates for bread wheat resistance to dwarf bunt, 0.88–0.93, (Chen et al. 2016) and common bunt, 0.58–0.78 (Bhatta et al. 2018; Mourad et al. 2018). Although the broad-sense heritability estimate and correlations between replications and years were high in this study, there were no significant SNPs that were consistent between data sets and met the FDR-adjusted *P* value threshold of 0.05 (Supplementary File 8). Less stringent significance thresholds have been used in other bread wheat GWAS panels with small population sizes (Zegeye et al. 2014; Gao et al. 2016). A less stringent threshold would allow identification of additional marker-trait associations in this panel (Supplementary File 7), but would increase the likelihood of false positive associations.

Of the two marker-trait associations that were significant in the present study (Table 5), only one corresponds with a previously reported QTL for DB or CB resistance (Supplementary File 1). Menzies et al. (2006) and Singh et al. (2016) found a QTL on 6DS with a peak marker at 6.17 Mbp, which is likely the same QTL identified as *DB*-*6D2* in this study. *DB*-*6D2* is composed of two SNP markers and is most significantly associated with resistance identified in the present study (Table 5). Accessions containing the resistance alleles had a mean DB NI BLUE value of 16.3 (Supplementary File8). The *Bt10* differential PI 178383 and another 30 accessions in this GWAS panel have this resistance-associated haplotype (Supplementary File 9). Based on the physical position, this QTL spanned a relatively narrow section of the chromosome from 6.97 to 7.29 Mbp, which is within the flanking position of the *Bt10* gene (Menzies et al. (2006). Markers in this region can be developed and used in marker-assisted selection, but must first be validated in biparental populations.

Additionally, Menzies et al. (2006) hypothesized that the *Bt10* QTL contributed by the bread wheat cultivar ‘AC Cadillac’ was closely linked with effective Ug99 stem rust resistance genes on 6DS, *SrTmp* or *SrCad* (Hiebert et al. 2016; Kassa et al. 2016). To determine whether *Bt10* confers a stem rust resistance phenotype like *SrCad* or *SrTmp*, PI 554118 (*Bt10*) and PI 178383 were screened with several Ug99 stem rust races. These two accessions were resistant to many of the same stem rust races as lines containing *SrTmp* and *SrCad* (unpublished data). Further studies are needed to determine if the 6DS region contains one or more genes that confer resistance to DB, CB, and wheat stem rust.

Two major QTL, *Q.DB.ui*-*7DS* (Chen et al. 2016), *Q.DB*-*6DL* (Steffan et al. 2017; Wang et al. 2019) and *Q.DB.ui*-*7AL* (Wang et al. 2019) that were previously reported in biparental populations, were not detected in this study. The QTL *Q.DB.ui*-*7DS* was reported in the ‘Rio Blanco’/’IDO444’ population on 7DS with a peak marker, *wPt*-*2565,* at 5.9 Mbp near the telomere (Chen et al. 2016). Based on pedigree information, the resistance in IDO444 was thought to be derived from PI 476212, the same parent contributing resistance in cv. ‘Blizzard.’ PI 476212 was initially selected for snow mold and DB resistance (Sunderman et al. 1986) and is in the pedigree of resistant cultivars ‘DW’ (PI 620629), ‘Bonneville’ (PI 557015), ‘Golden Spike’ (PI 614813), and ‘UI Silver’ (PI 658467). PI 476212 was highly resistant in the present study and was 99.99% similar to PI 173438 (unknown *Bt*) across the 19,281 SNPs, but the 7DS QTL reported by Chen et al. (2016) was not detected, possibly because too few accessions with this QTL were included in the present study. A haplotype analysis using SNPs in the 7DS region indicated that three of the highly resistant landraces in addition to the *Bt12* differential and PI 476212 may contain the 7DS QTL (Table 6, Supplementary File 9).

Similarly, *Bt9* has been mapped to 6DL between 172.8 and 175.9 Mbp in a population derived from the *Bt9* differential PI 554099 (Steffan et al. 2017). However, our GWAS did not detect any markers significantly associated with 6DL in any of the data sets. Using a biparental mapping population derived from a University of Idaho wheat breeding line ‘IDO835’, Wang et al. (2019) found two QTL for DB resistance, one on 6DL corresponding with the *Bt9* locus, and one on 7AL. We used the resistant haplotypes for both loci to find accessions that contain these QTL (Table 6, Supplementary File 9). The *Bt9* differential and PI 178383 contained the haplotype profile for the 6DL locus, but none of the highly resistant accessions contained the 6DL or 7AL haplotype (Table 6, Supplementary File 9).

Aside from the possible low frequency of certain known loci in our GWAS panel, SNP maker filtering could also have reduced detection of known loci. SNP markers were filtered at a MAF threshold of 4% and any marker with fewer than thirteen individuals in each allelic state would have been filtered before analysis. This filtering threshold could mask SNP-trait associations that were present at low frequencies. To find such QTL, biparental populations could be developed with resistant accessions from the panel that lack alleles for the previously identified QTL.

Several marker-trait groups were associated with specific subpopulations (Supplementary File 11). For instance, 48% of accessions with the *QDB*-*6D2*-resistant haplotype are in subpopulation 4 (Supplementary Files 9 and 11). All the highly resistant accessions and 12 of the bunt differentials contained the *DB*-*6D1* haplotype group. Conversely, the resistant haplotype for *DB*-*6D2* was strongly associated resistance (Table 5); however, none of the highly resistant landrace accessions contained this haplotype (Table 6, Supplementary File 9).

The present study evaluated the DB responses recorded in the GRIN database for 292 wheat accessions rated in three field trials and identified 98 accessions that were resistant and 28 accessions that were highly resistant across all three years of USU field trials and in GRIN. Additionally, four SNP markers associated with DB resistance were identified, one marker-trait association group on 6D was consistent across several data sets, and one marker-trait association group on chromosome 6D was not previously reported. Of the highly resistant landrace accessions, six have novel resistance haplotype profiles. These resistant accessions and haplotype regions can be used to confirm resistance loci in biparental mapping populations for introgression into advanced wheat breeding lines.

## Electronic supplementary material

Below is the link to the electronic supplementary material.

Supplementary material 1 (DOCX 18 kb)

Supplementary material 2 (XLSX 59 kb)

Supplementary material 3 (DOCX 12 kb)

Supplementary material 4 (PPTX 113 kb)

Supplementary material 5 (PPTX 120 kb)

Supplementary material 6 (PPTX 78 kb)

Supplementary material 7 (XLSX 3885 kb)

Supplementary material 8 (XLSX 11 kb)

Supplementary material 9 (XLSX 38 kb)

Supplementary material 10 (XLSX 9 kb)

Supplementary material 11 (XLSX 9 kb)
