# Supplementary material for: Genetic characterization and genome-wide association mapping for dwarf bunt resistance in bread wheat accessions from the USDA National Small Grains Collection
Source: Theor Appl Genet. 2020 Jan 14;133(3):1069–80. doi: 10.1007/s00122-020-03532-0 (PMC7021738; doi:10.1007/s00122-020-03532-0)
Supplement: Supplementary file 1 — Supplementary material 1 (DOCX 18 kb) [file 122_2020_3532_MOESM1_ESM.docx]

Genetic characterization and genome-wide association mapping for dwarf bunt resistance in bread wheat accessions from the USDA National Small Grains Collection

**Supplementary File 1** Previously reported common bunt (CB) and dwarf bunt (DB) resistance loci

| Chromosome | Position (cM) | Disease | Resistance Source | Reference |
| --- | --- | --- | --- | --- |
| 1A | 74-76 | DB CB | IDO444; PI 476212 | Chen et al. (2016); Mourad et al. (2018) |
| 1BS | 19-20 | CB | ‘AC Domain’; ‘Blizzard’; PI 476212 | Fofana et al. (2008); Wang et al. (2009) |
| 1BS | 45 | CB | ‘Trintella’ | Dumalasová et al. (2012) |
| 1BS | 43.8 | CB | ‘Carberry’ | Singh et al. (2016) |
| 1BS | 47-55 | CB | ‘CDC Go’ | Zou et al. (2017) |
| 1B | 76-96 | CB | ‘AC Domain’ | Fofana et al. (2008); Bhatta et al. (2018); Mourad et al. (2018) |
| 2A |  | CB |  | Bhatta et al. (2018) |
| 2BS | 13-15.2 | DB | IDO444; PI 476212 | Chen et al. (2016) |
| 2BL |  | CB |  | Bhatta et al. (2018); Mourad et al. (2018) |
| 3AL | 200-206 | CB | ‘CDC Go’ | Zou et al. (2017); Mourad et al. (2018) |
| 3BS |  | CB |  | Mourad et al. (2018) |
| 3DL |  | CB |  | Bhatta et al. (2018) |
| 4AL |  | CB |  | Bhatta et al. (2018); Mourad et al. (2018) |
| 4BS | 62.3-89.4 | CB | ‘Carberry’ | Singh et al. (2016) |
| 4DS | 7.44 | CB | ‘Carberry’ | Singh et al. (2016) |
| 5AL |  | CB |  | Mourad et al. (2018) |
| 5BS | 0 | CB | ‘Trintella’ | Dumalasová et al. (2012) |
| 5BL |  | CB |  | Mourad et al. (2018) |
| 5DL |  | CB |  | Mourad et al. (2018) |
| 6AL |  | CB |  | Mourad et al. (2018) |
| 6BL |  | CB |  | Mourad et al. (2018) |
| 6DS | 9.7 | CB | ‘AC Cadillac’; ‘AC Taber’; PI 178383 | Menzies et al. (2006); Singh et al. (2016) |
| 6DL | 125-132 | CB | PI 178383; PI 554099 | Steffen et al. (2017) |
| 7AS | 44 | CB | ‘Trintella’ | Dumalasová et al. (2012); Bhatta (2018) |
| 7AL | 117-120 | CB | ‘AC Domain’ | Fofana et al. (2008); Bhatta et al. (2018); Mourad et al. (2018) |
| 7BS | 9.6 | CB | ‘McKenzie’ | Knox et al. (2013) |
| 7BS | 13 | CB | ‘Trintella’ | Dumalasová et al. (2012); Mourad et al. (2018) |
| 7BL |  | CB |  | Bhatta et al. (2018); Mourad et al. (2018) |
| 7DS | 1 | DB | IDO444; PI 476212 | Chen et al. (2016) |
| 7DL | 46.6 | CB | ‘Carberry’ | Singh et al. (2016) |
| 7DL |  | CB |  | Mourad et al. (2018) |
