# Supplementary material for: Genetic characterization and genome-wide association mapping for dwarf bunt resistance in bread wheat accessions from the USDA National Small Grains Collection
Source: Theor Appl Genet. 2020 Jan 14;133(3):1069–80. doi: 10.1007/s00122-020-03532-0 (PMC7021738; doi:10.1007/s00122-020-03532-0)
Supplement: Supplementary file 3 — Supplementary material 3 (DOCX 12 kb) [file 122_2020_3532_MOESM3_ESM.docx]

**Supplementary File 3** Restricted maximum likelihood variance component estimates and significance values for 292 wheat accessions across four trials

| Effect | Variance ratio | Variance component | Standard error | *P*-value | Percent |
| --- | --- | --- | --- | --- | --- |
| Accession | 5.02 | 1601.08 | 141.43 | <.0001 | 74.91 |
| Trial | 0.24 | 76.21 | 63.32 | 0.2287 | 3.57 |
| Accession*Trial | 0.44 | 141.21 | 27.76 | <.0001 | 6.61 |
| Residual |  | 318.75 | 21.97 |  | 14.91 |
| Total |  | 2137.25 | 155.86 |  | 100 |
