# Supplementary material for: Genetic characterization and genome-wide association mapping for dwarf bunt resistance in bread wheat accessions from the USDA National Small Grains Collection
Source: Theor Appl Genet. 2020 Jan 14;133(3):1069–80. doi: 10.1007/s00122-020-03532-0 (PMC7021738; doi:10.1007/s00122-020-03532-0)
Supplement: Supplementary file 4 — Supplementary material 4 (PPTX 113 kb) [file 122_2020_3532_MOESM4_ESM.pptx]

## Slide 1
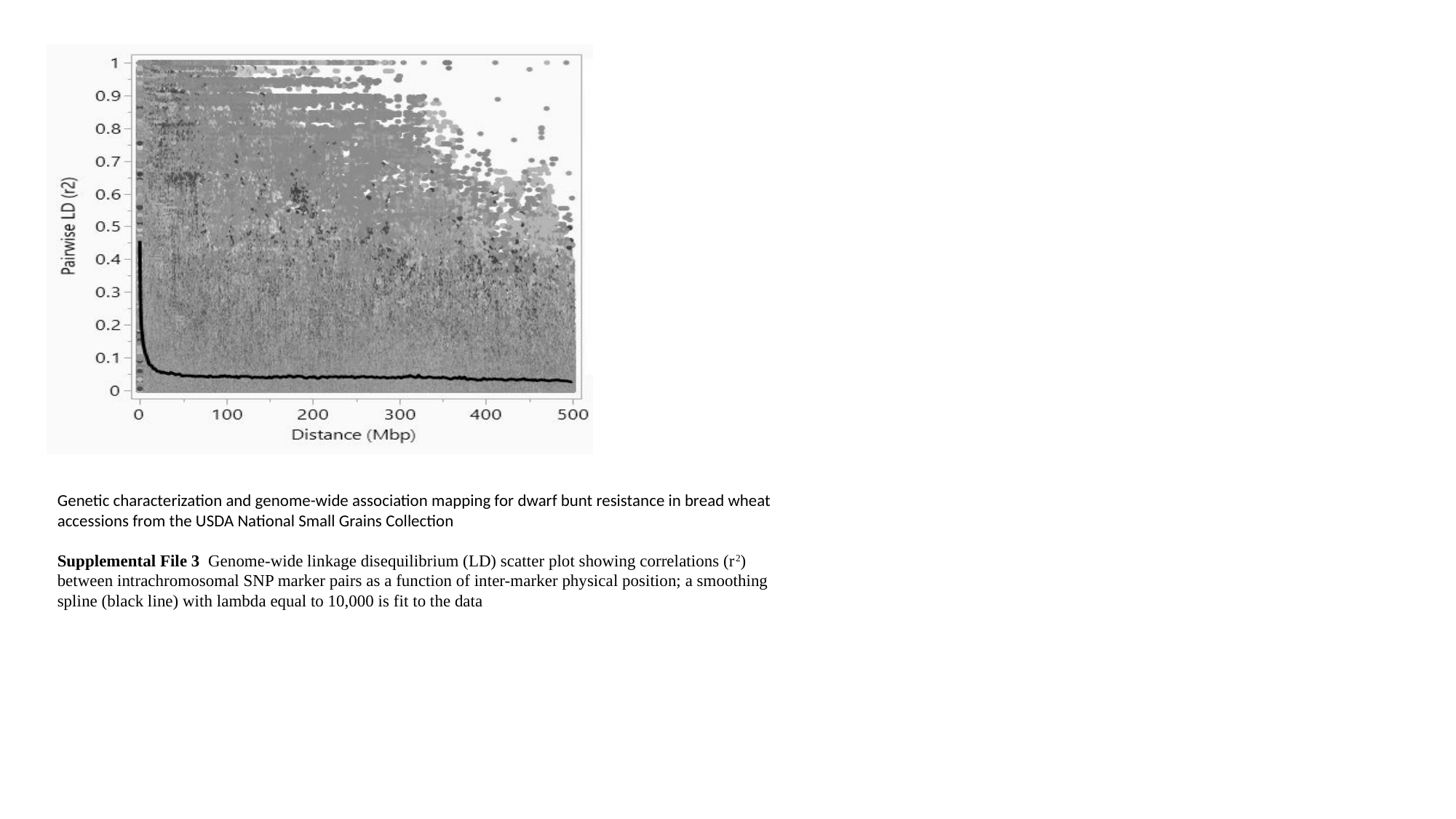

Genetic characterization and genome-wide association mapping for dwarf bunt resistance in bread wheat accessions from the USDA National Small Grains Collection
Supplemental File 3 Genome-wide linkage disequilibrium (LD) scatter plot showing correlations (r2) between intrachromosomal SNP marker pairs as a function of inter-marker physical position; a smoothing spline (black line) with lambda equal to 10,000 is fit to the data
