# Supplementary figures and images for: Genetic characterization and genome-wide association mapping for dwarf bunt resistance in bread wheat accessions from the USDA National Small Grains Collection
Source: Theor Appl Genet. 2020 Jan 14;133(3):1069–80. doi: 10.1007/s00122-020-03532-0 (PMC7021738; doi:10.1007/s00122-020-03532-0)

## Slide 1
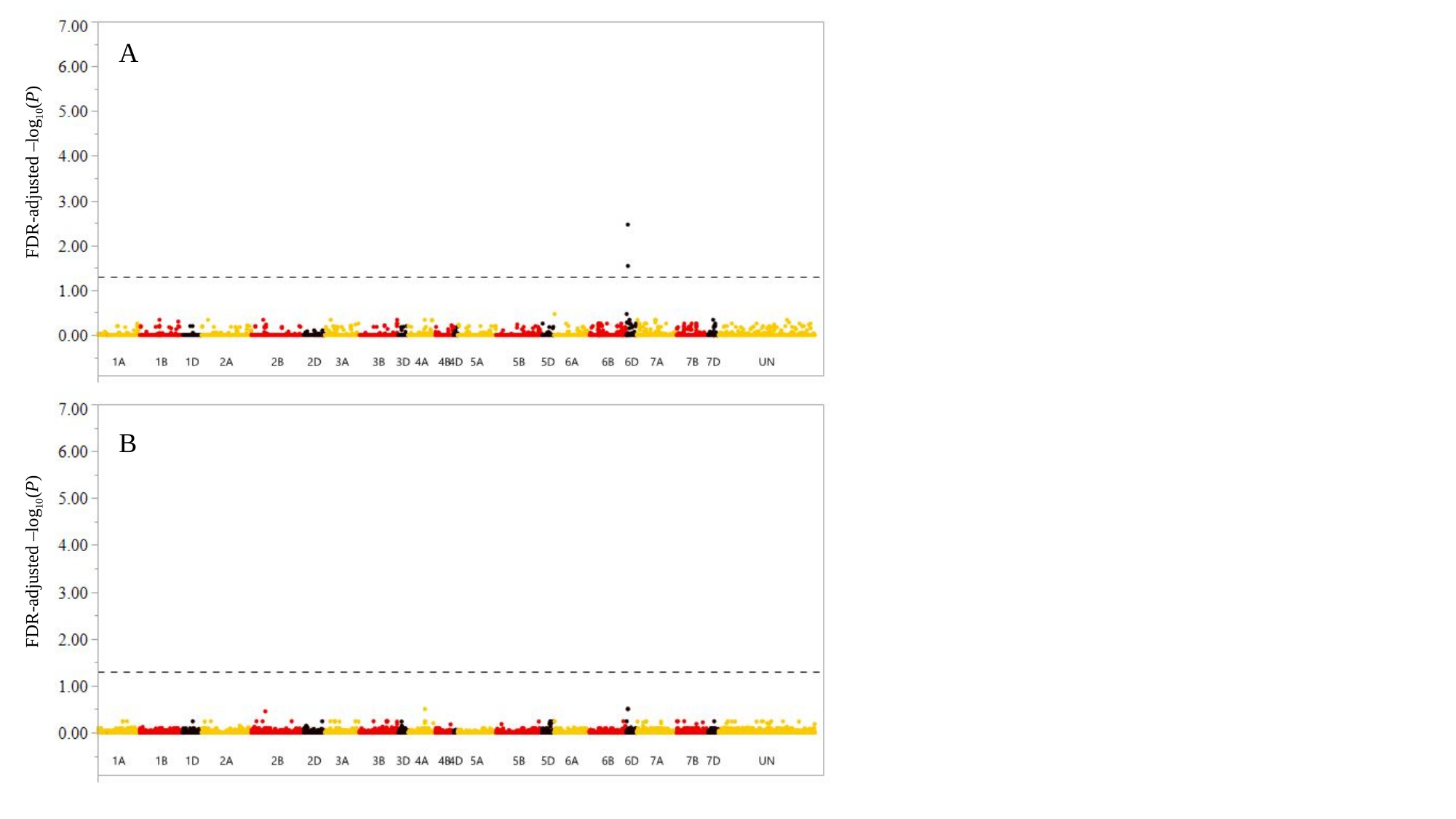

A
FDR-adjusted –log10(P)
B
FDR-adjusted –log10(P)

## Slide 2
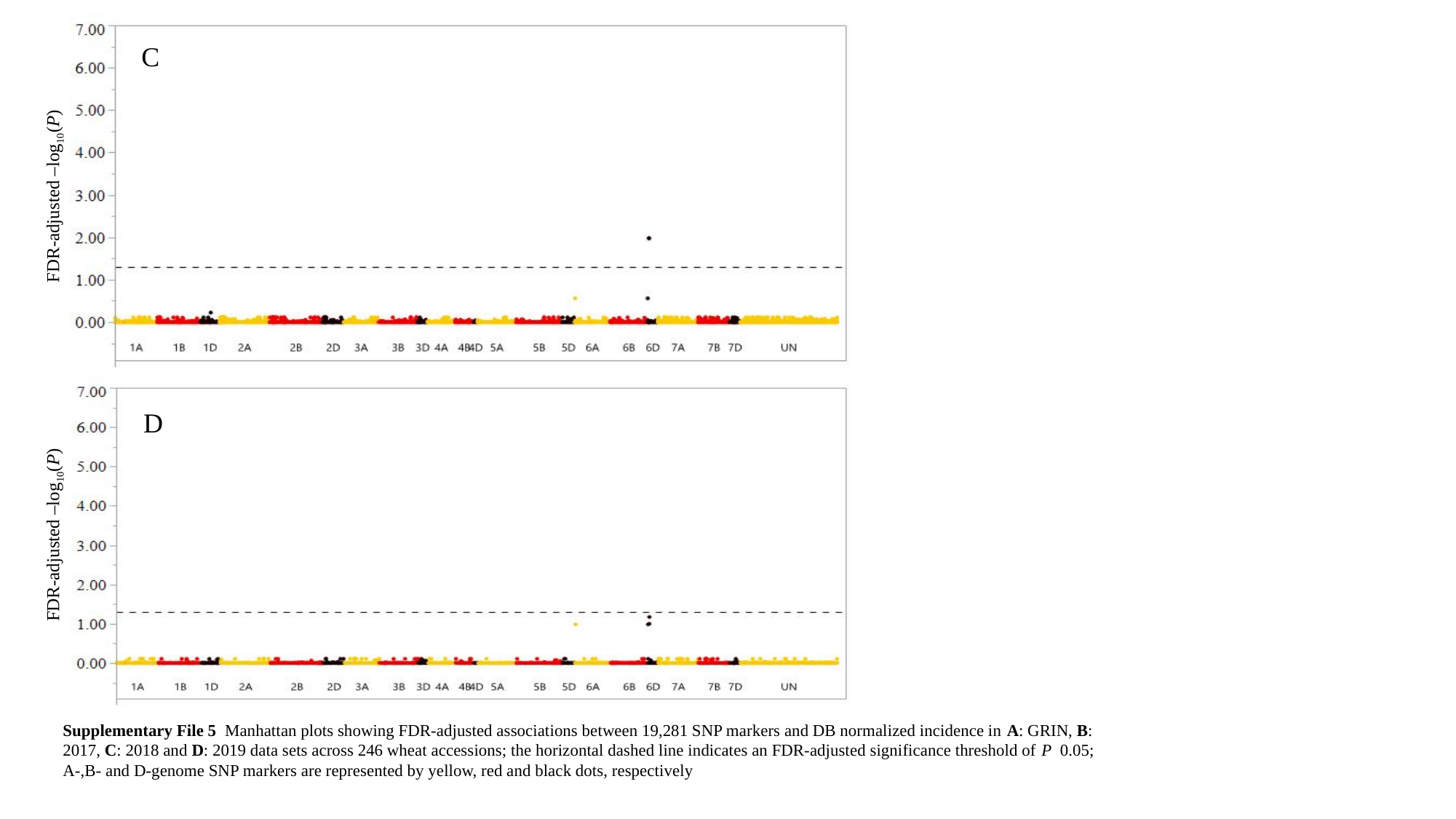

C
FDR-adjusted –log10(P)
D
FDR-adjusted –log10(P)

Supplement: Supplementary file 5 — Supplementary material 5 (PPTX 120 kb) [file 122_2020_3532_MOESM5_ESM.pptx]
