# Supplementary material for: Genetic characterization and genome-wide association mapping for dwarf bunt resistance in bread wheat accessions from the USDA National Small Grains Collection
Source: Theor Appl Genet. 2020 Jan 14;133(3):1069–80. doi: 10.1007/s00122-020-03532-0 (PMC7021738; doi:10.1007/s00122-020-03532-0)
Supplement: Supplementary file 6 — Supplementary material 6 (PPTX 78 kb) [file 122_2020_3532_MOESM6_ESM.pptx]

## Slide 1
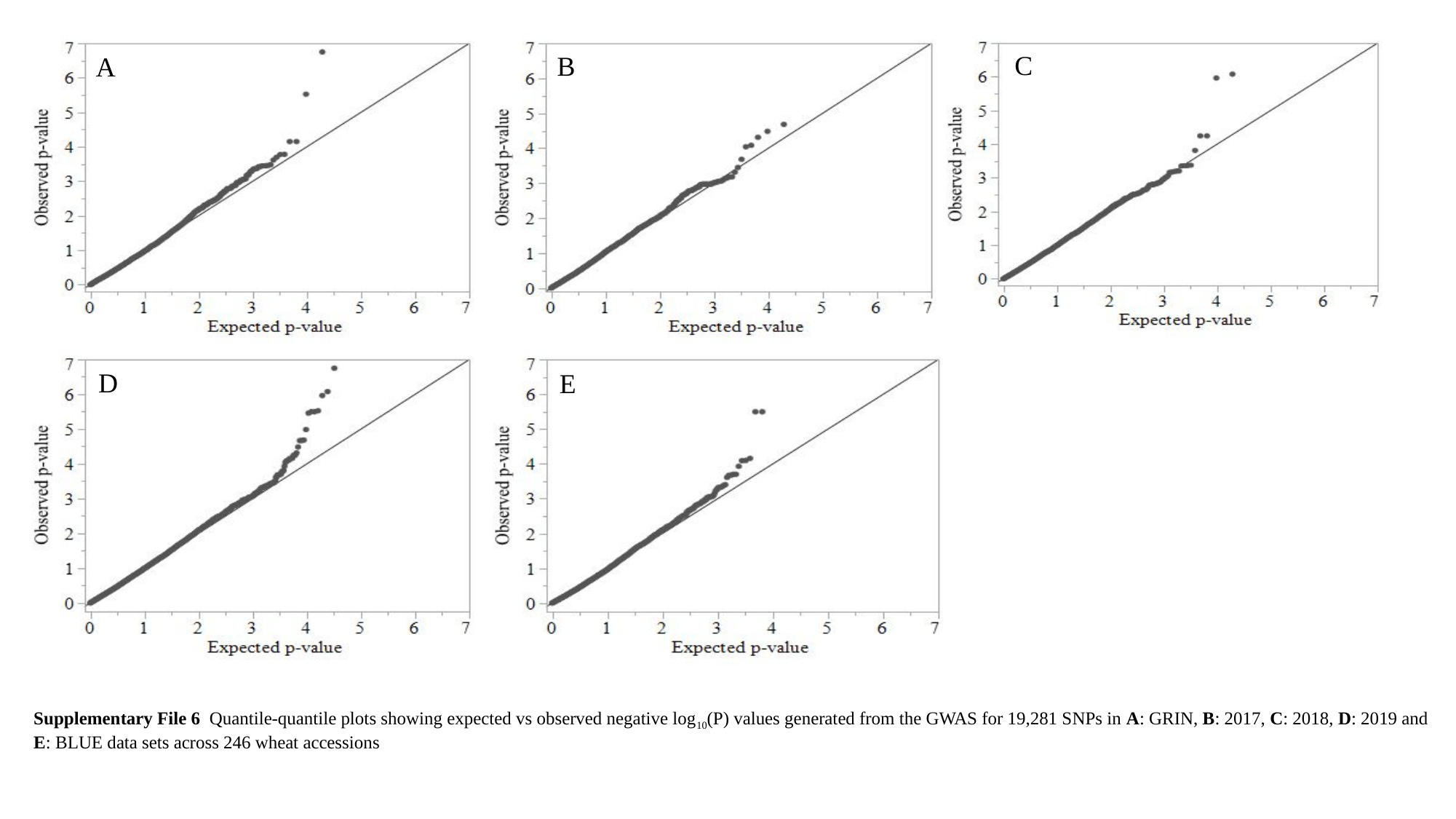

C
B
A
D
E
Supplementary File 6 Quantile-quantile plots showing expected vs observed negative log10(P) values generated from the GWAS for 19,281 SNPs in A: GRIN, B: 2017, C: 2018, D: 2019 and E: BLUE data sets across 246 wheat accessions
